# A quality by design approach for the synthesis of palmitoyl-L-carnitine-loaded nanoemulsions as drug delivery systems

**DOI:** 10.1080/10717544.2023.2179128

**Published:** 2023-02-19

**Authors:** E. M. Arroyo-Urea, María Muñoz-Hernando, Marta Leo-Barriga, Fernando Herranz, Ana González-Paredes

**Affiliations:** a Nanomedicine and Molecular Imaging group, Instituto de Química Médica-CSIC, Madrid, Spain; b Centro de Investigación Biomédica en Red de Enfermedades Respiratorias (CIBERES), Madrid, Spain; c Conexión Nanomedicina-CSIC, Madrid, Spain

**Keywords:** Nanoemulsion, drug delivery, design of experiments, colloidal stability, biodistribution

## Abstract

Nanoemulsions (NE) are lipid nanocarriers that can efficiently load hydrophobic active compounds, like palmitoyl-L-carnitine (pC), used here as model molecule. The use of design of experiments (DoE) approach is a useful tool to develop NEs with optimized properties, requiring less experiments compared to trial-and-error approach. In this work, NE were prepared by the solvent injection technique and DoE using a two-level fractional factorial design (FFD) as model was implemented for designing pC-loaded NE. NEs were fully characterized by a combination of techniques, studying its stability, scalability, pC entrapment and loading capacity and biodistribution, which was studied *ex-vivo* after injection of fluorescent NEs in mice. We selected the optimal composition for NE, named pC-NE_U_, after analysis of four variables using DoE. pC-NE_U_ incorporated pC in a very efficient manner, with high entrapment efficiency (EE) and loading capacity. pC-NE_U_ did not change its initial colloidal properties stored at 4 °C in water during 120 days, nor in buffers with different pH values (5.3 and 7.4) during 30 days. Moreover, the scalability process did not affect NE properties and stability profile. Finally, biodistribution study showed that pC-NE_U_ formulation was predominantly concentrated in the liver, with minimal accumulation in spleen, stomach, and kidneys.

## Graphical Abstract

**Figure:**
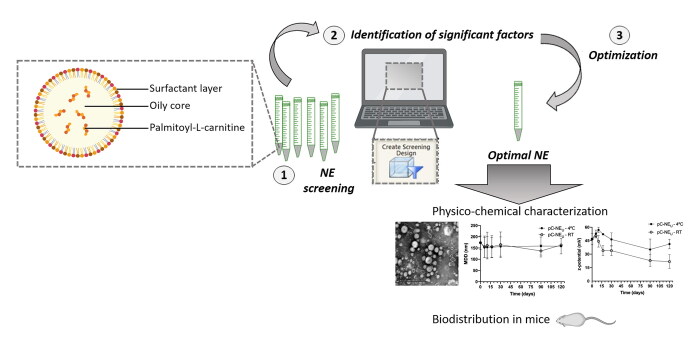

## 1. Introduction

Drug delivery nanosystems are designed technologies that allow drugs to be transported in a controlled manner. Because of their usefulness in modulating drug release, protecting labile materials (e.g. peptides, DNA, or mRNA) against degradation, and site-specific drug targeting, research in nanoparticles as drug vehicles has been a very active field for many years. This activity has been boosted by the success in the use of lipid nanoparticle-based vaccines (Thi et al., 2021; Mitchell et al., 2021). Compared with other carriers, such as polymeric nanoparticles, lipid-based nanoparticles are gaining attention in the pharmaceutical industry thanks to their biocompatibility and formulation simplicity, making them the most promising vehicles to deliver a variety of therapeutics (Lu et al., 2021; Dhiman et al., 2021).

Among the different lipid-based nanoplatforms, nanoemulsions (NE), constituted by oil, water, and surfactants and with droplet sizes ranging from 10 to 1000 nm, are gaining attention for the delivery of hydrophobic drugs (Sánchez-López et al., 2019; Guzmán et al., 2021). Considerable research is nowadays ongoing to encapsulate hydrophobic drugs in order to improve their bioavailability, safety, and efficacy (Yang et al., 2019; Louage et al., 2017; Klein et al., 2020). Nevertheless, developing a versatile and controllable drug encapsulation system with high drug loading still remains a challenge. For the various nanoparticulated systems reported, drug loading is usually below 10 % or even 1% (Liu et al., 2020). Due to their recognized ability for facilitating the encapsulation of the hydrophobic molecules through the lipid matrix, NE have been used to wrap essential oils and nutrients, and a large number of studies have reported using them to package different types of drugs, such as paclitaxel (Shakhwar et al., 2020), curcumin (Prasad et al., 2020), and retinoic acid (Tinoco et al., 2018), among others.

Pharmaceutical industry is implementing quality by design (QbD) approaches motivated by the stringent need of ensuring products safety, quality, and efficacy (Jain, 2014). Considering QbD, product quality can be controlled by the identification of critical factors –independent variables – and their influence in obtained responses – dependent variables – allowing the optimization of the process. The QbD approach involves statistical design of experiments (DoE) which allows to identify the relationships between the factors influencing a process and the observed outputs, and helping in the identification of optimal process conditions within the space of the design (González-Fernández et al., 2021). Compared to trial-and-error and one-factor-at-time (OFAT) approaches, DoE provides more information from datasets, allowing minimization of experimental efforts for a given statistical power and giving the possibility of working with different type of constrains (Lee, 2019). Furthermore, a thorough understanding of these processes is essential for later scale-up and quality control as needed for preclinical and clinical test batches. Although DoE has several advantages in the rational design of lipid nanoparticles, its use is still scarce in the literature. Nevertheless, the most frequently evaluated responses are particle size, polydispersity index (PDI), zeta potential, drug loading, and EE, as these are parameters that highly influence particles stability and biological behavior, whereas the studied factors influencing these responses are related to lipids and surfactants composition and conditions for synthesis (Tavares Luiz et al., 2021).

Our aim was to use QbD approach to develop an O/W NE with optimal physicochemical and colloidal stability properties as drug carrier for encapsulation of hydrophobic drugs, using palmitoyl-L-carnitine (pC) as model molecule. Different types of experimental designs can be used, and in this work, a fractional factorial design (FFD) is proposed as it is a rapid and reliable tool, allowing the exploration of a maximum number of variables requiring less experimental observations than full factorial without a lack of main effects data (Kuncahyo et al., 2019). pC, the selected model molecule, is an organic compound containing a long-chain acyl fatty acid attached to carnitine through an ester bond. Its low solubility in water (1.2e^−05^ g/L) makes it an excellent candidate to be used as a model hydrophobic therapeutic drug. As an active compound several biological activities have been described for pC: capability of altering the activity of various enzymes and transporters found in human membrane cells (Bernatoniene et al., 2011), prevention of biofilm formation in *Escherichia coli* and *Pseudomonas aeruginosa* (Wenderska et al., 2011) and activation of sphingosine-1-phosphate (S1P) receptors (S1PRs), which are becoming more widely recognized as important regulators of homeostasis and disease for their role in cell survival, activation status and proliferation in all biological systems (Blaho & Hla, 2014).

## 2. Materials

Polysorbate 80 (T80) (MW 428.6) and polysorbate 20 (T20) (MW 604.813) were kindly donated by Croda Iberica (Barcelona, Spain). pC (MW 399.61) (≥97% HPLC), (±)-α-Tocopherol (TOC) (MW 430.71) (≥96% HPLC), Octadecylamine (ODA) (MW 269.51), Phosphate-buffered saline (PBS) and D-mannitol, as well P10 desalting columns (bed size 14.5 mm × 50 mm) were purchased from Sigma-Aldrich (Madrid, Spain). DiD’ oil (1,1′-Dioctadecyl-3,3,3′,3′-Tetramethylindodicarbocyanine Perchlorate) (MW 959.9) was purchased from Fisher Scientific (Madrid, Spain). Acetic acid (Scharlab, Barcelona, Spain), potassium hydroxide (Sigma-Aldrich, Madrid, Spain), potassium phosphate monobasic (Sigma-Aldrich, Madrid, Spain), and sodium phosphate dibasic (Sigma-Aldrich, Madrid, Spain) were used to prepare buffers of different pH. Vivaflow^®^ 50 Cassettes (Regenerated Cellulose, 100 KDa) and 0.2 µm filters (Regenerated Cellulose) were purchased from Sartorius Stedim Biotech (Göttingen, Germany). Acetonitrile HPLC Supragradient was purchased from Scharlab (Barcelona, Spain). Other chemicals were of analytical reagent grade and used without further purification.

## 3. Methods

### 3.1. Surfactant screening

The initial aim was to select the best surfactant for NE formation, thus two non-ionic surfactants, T80 and T20, were evaluated for their ability to form stable free-drug NE (Blank NE). The method used for preparation of NE was the solvent injection technique reported earlier (Schubert & Müller-Goymann, 2003). Briefly, an organic phase with 250 µL of ethanol containing TOC (5 mg), ODA (1 mg), and different amounts of surfactant (0.1 mg, 0.5 mg, 5 mg, and 10 mg) was prepared. The organic phase was then injected into 1 mL of PBS solution (PBS 1x) at RT under continuous stirring (700 rpm) in order to form the NE. The resulting NE were purified by size exclusion chromatography using P10 desalting columns. These columns contain Sephadex G-25 resin, which allows the removal of solvent and non-incorporated excipients. Briefly, 2.5 mL of NE was loaded in the column and the purified NE was eluted with 3.5 mL of fresh PBS or ultrapure water using gravity flow.

### 3.2. Identification of critical variables by QbD approach

After selecting the best surfactant, a QbD approach using DoE was implemented for screening and designing pC-loaded NE (pC-NE) (Design Expert version 12.1). A two-level FFD (resolution IV) was selected as model: four independent variables (final concentration of TOC, ODA, T80, and pC) and three dependent variables (hydrodynamic diameter, PDI, and zeta potential) were defined and levels for each factor were established (Table 1), with addition of 3 center points used to test for curvature. The selected model required the preparation of nineteen pC-NE, which were synthesized by the addition of pC to 250 µL of ethanol solution containing the other components, as described for the synthesis of blank NE in 3.1.

**Table 1.** Selected variables levels for pC-NE screening. The experimental levels (low and high) are represented by the coded values of −1 and +1, respectively, corresponding to the final concentration (mg/mL) of the components.

		Level	
Independent variables	Variable codification	−1	+1
TOC	A	2.5	7.5
ODA	B	0.15	0.85
pC	C	0.5	1.5
T80	D	0.25	1.75

Later, the obtained data were analyzed using Pareto charts and half-normal plots, followed by ANOVA analysis, in order to identify the factors exhibiting the highest influence on the chosen critical quality attributes. Finally, the analysis of obtained results using DoE allowed us to predict two optimal pC-NE compositions according to the desired physicochemical properties, which were further developed and studied (pC-NE_T_ and pC-NE_U_ formulations).

### 3.3. Physicochemical characterization of NE

#### 3.3.1. Particle size characterization

The hydrodynamic diameter and PDI of blank NE and pC-NE were assessed by photon correlation spectroscopy (PCS) using a Zetasizer nano ZS90 (Malvern Panalytical, Malvern, UK). All measurements were performed in triplicates at RT.

#### 3.3.2. Determination of zeta potential

The surface charge of pC-NE was determined by Electrophoretic Light Scattering (ELS) using a Zetasizer Nano ZS90 (Malvern Panalytical, Malvern, UK). The nanosuspensions synthesized in PBS were diluted (1:10) in ultrapure water prior to zeta potential analysis, whereas those synthesized in ultrapure water were diluted (1:20) in sodium chloride 1 mM prior to zeta potential analysis. Each sample was analyzed in triplicates at RT.

#### 3.3.3. Morphology determination by transmission electron microscopy

The morphology of pC-NE (formulations pC-NE_T_ and pC-NE_U_) was examined by transmission electron microscopy (TEM). TEM analyses were performed at the National Center of Electronic Microscopy of the Complutense University of Madrid (Madrid, Spain). NE were placed on the surface of carbon-coated copper grids, negatively stained with 2% uranyl acetate and observed under TEM using a JEOL JEM 1400 instrument operated at 100 kV equipped with a CCD camera Gatan Orius Sc 200.

### 3.4. Stability studies

Both pC-NE_T_ and pC-NE_U_ formulations were subjected to stability studies in triplicate. The physical stability in PBS of both NE was evaluated following storage at 4 °C and RT, by measurement of hydrodynamic diameter, PDI, and zeta potential at pre-established time-points during 2 weeks. The behavior of pC-NE_U_ formulation in ultrapure water at 4 °C and RT and at different pH conditions (pH 7.4 and pH 5.3) was also investigated up to 4 months.

### 3.5. Determination of entrapment efficiency of pC-NE

The concentration of pC in pC-NE_U_ formulation was determined before and after purification process through HPLC-RID (Agilent 1260 system) analysis using a Waters Symmetry C18 column (4.6 × 75 3.5um). The chromatography was carried out at a flow rate of 1 mL/min in isocratic conditions, using acetonitrile/KH_2_PO_4_ (50 mM) (65:35 v/v) at pH = 3.5 adjusted with o-phosphoric acid as mobile phase. For NE disruption and sample preparation see Supplementary Material. *EE* was calculated using the following formula:

$$EE(\%)=\frac{C_{AP}}{C_{BP}}\times 100$$

where *C_AP_* is the pC concentration found in disrupted NE after the purification process and *C_BP_* is the pC concentration found in disrupted NE before the purification process.

### 3.6. Determination of reaction yield and loading capacity of pC-NE

For determining the yield of the synthesis process resulting in NE formation, NE_U_ formulations (both blank and pC-NE_U_) were synthesized as previously described and resulting purified formulations were lyophilized and accurately weighted to obtain the total mass of NE (m*_exp_*). The reaction yield (*R*) was calculated as follows:

$$R(\%)=\frac{m_{\exp}}{m_{\mathrm{the}}}\times 100$$

where m*_exp_* is the total mass of NE found after the purification process and m*_the_* is the theoretical total mass of NE.

The loading capacity (*LC*) in pC-NE_U_ was calculated using the EE as follows:

$$LC(\%)=\frac{EE\times \mathrm{total}\;pC\;\mathrm{amount}}{m_{\exp}}\times 100$$

Determinations in 3.5 and 3.6 were done in triplicate.

### 3.7. pC-NE_U_ formulation biodistribution

For biodistribution studies, pC-NE_U_ was synthesized in mannitol (5.5% w/v) and loaded with DiD’ oil, a dialkylcarbocyanine with markedly red-shifted fluorescence excitation and emission spectra. Due to its remarkable lipophilic nature, DID’ oil was added in the organic phase during the synthesis procedure. The molar ratio between pC and DID’ oil was 1:0.02. The fluorescent formulation was characterized by measuring hydrodynamic size, PDI, and zeta potential, as previously described. The EE of the dye was determined spectrophotometrically using a Clariostar microplate reader using the same formula than for pC EE (see Section 3.5). Biodistribution studies were performed on healthy mice. For that purpose, four C57BL/6 mice were intravenously injected with the fluorescent pC-NE_U_ formulation (DiD-pC-NE_U_). In addition, one C57BL/6 mouse was left non-injected as control. Four hours post injection, mice were sacrificed by carbon dioxide inhalation, perfused using 4% formaldehyde and dissected. Dissected tissues, including brain, heart, lungs, stomach, spleen, liver, bladder, kidneys, muscle, and bone were then analyzed using an *ex vivo* fluorescence imaging system. Detected fluorescence signal was adjusted until no fluorescence was present in the organs of the control animal, which allows visualizing the signal corresponding to NE, avoiding tissues autofluorescence. Animal experiments were conducted according to Spanish and EU regulations (PROEX277/16).

## 4. Results

### 4.1. Effect of surfactants on nanoemulsions characteristics

We studied the effect of two surfactants, T20 and T80, on particle size and distribution. T80 led to smaller particle sizes for all the tested concentrations compared to T20, as shown in Table 2. Increase in T20 concentration from 0.1 to 10 mg/mL caused a drastic particle size decrease from more than 700 nm to about 250 nm for NE (F5-F8). Moreover, increasing T80 concentration (F2-F4) resulted in slightly larger particles and higher polydispersity.

**Table 2.** Effect of surfactants polysorbate 80 (T80) and 20 (T20) concentration on the particle size and polydispersity of NE (mean ± SD, *n* = 3).

Lipid	Sample code	T80 (mg/mL)	T20 (mg/mL)	Mean particle size (nm)	PDI
TOC ODA	F1	0.1	–	304 ± 4	0.274 ± 0.014
	F2	1	–	240 ± 4	0.200 ± 0.013
	F3	5	–	255 ± 4	0.280 ± 0.021
	F4	10	–	279 ± 3	0.504 ± 0.036
	F5	–	0.1	700 ± 5	0.302 ± 0.040
	F6	–	1	320 ± 3	0.200 ± 0.024
	F7	–	5	295 ± 4	0.230 ± 0.017
	F8	–	10	246 ± 4	0.370 ± 0.023

### 4.2. Analysis of critical variables by DoE

A FFD with resolution IV was selected as model ($2_{IV}^{4-1}$). The power of the model was 95.3% for all evaluated effects, ensuring its suitability, and it is adjusted to a general linear model and its double interactions. It required the synthesis and characterization of nineteen formulations (for composition and physicochemical characteristics see Table S1 and Figure S1, Supplementary Material).

**Figure 1.**
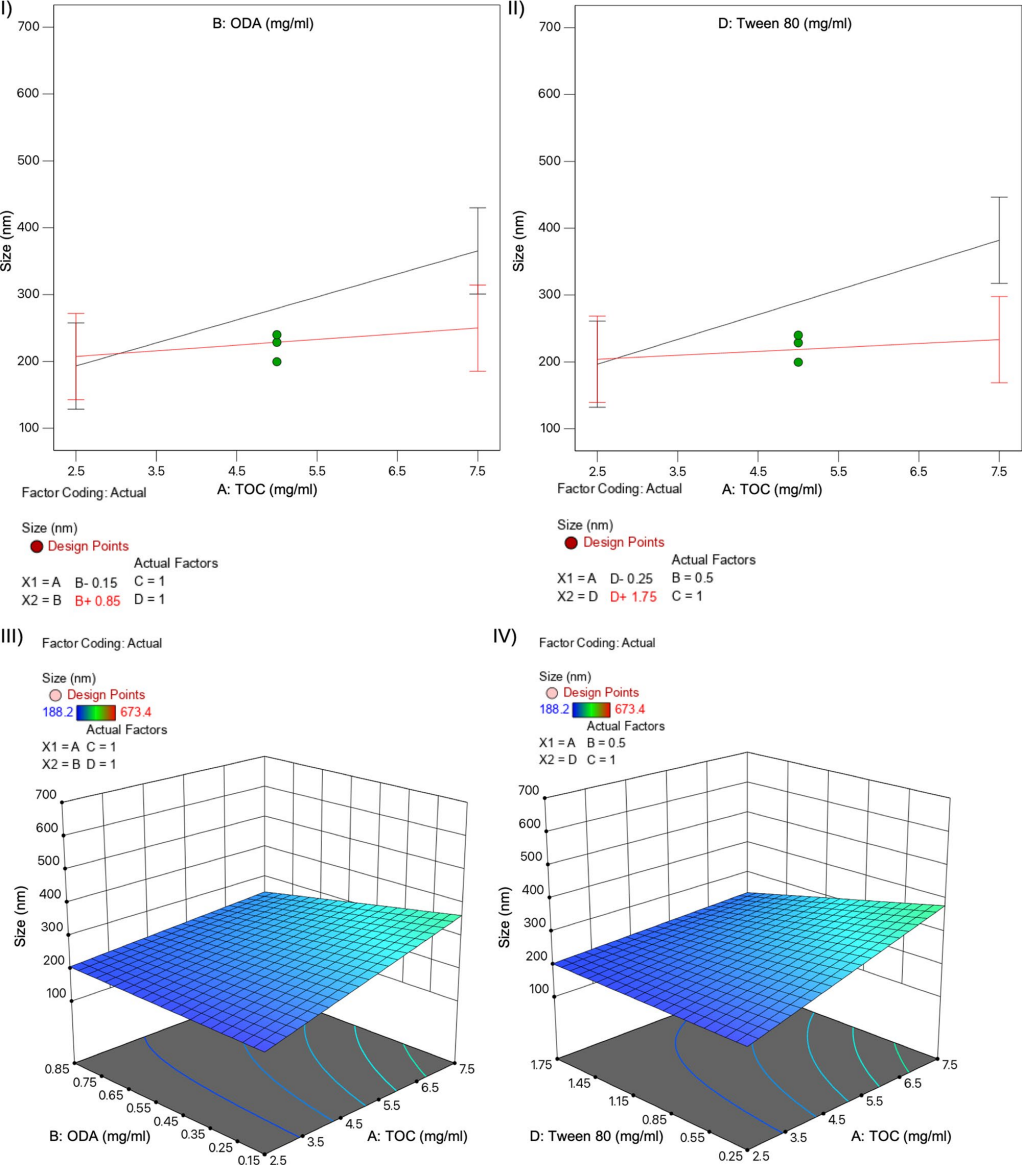
Normal plots (I-II) and 3D surface diagram (III-IV) for variable interactions influencing particle size: TOC-ODA (I and III) and TOC-T80 (II and IV).

#### 4.2.1. Effect of independent variables on size

Analysis of variance showed that the model was significant (F-value = 3.29) with a lack of fit not significant relative to the pure error. Among the evaluated independent variables, only TOC content (variable A) was statistically significant in influencing the particle size (*p* value=0.02). An increase in the oil amount is associated with the increase of particle size, which can be compensated with the addition of higher amount of the surfactant (T80) and/or the co-surfactant (ODA), as showed in the diagram for these interactions displayed in Figure 1 (I and II, red lines), so the interaction of variables AD (TOC-T80) and AB (TOC-ODA) influenced the size in somehow.

#### 4.2.2. Effect of independent variables on polydispersity index (PDI)

**Figure 2.**
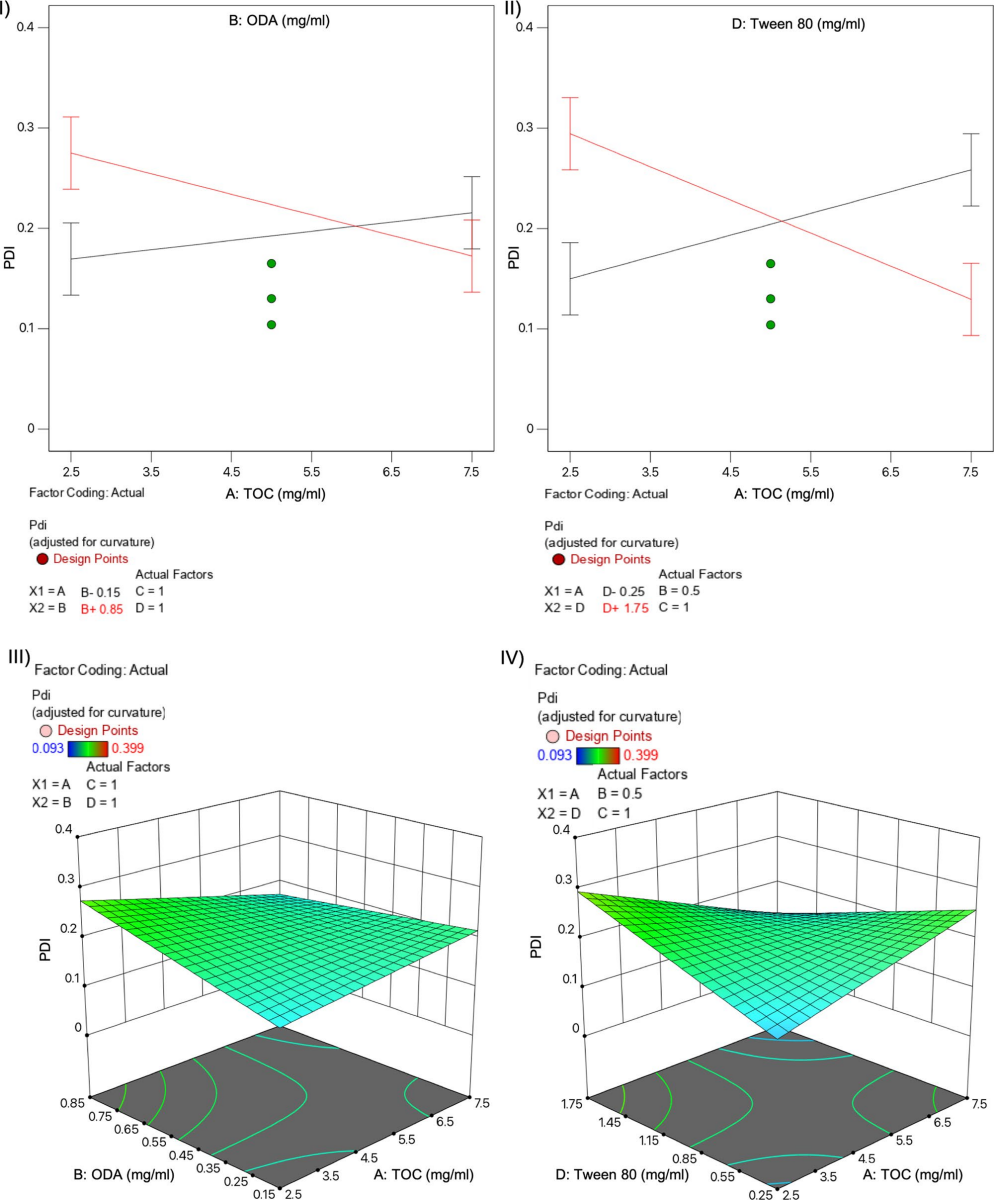
Normal plots (I-II) and 3D surface diagram (III-IV) for variable interactions influencing polydispersity index: TOC-ODA (I and III) and TOC-T80 (II and IV).

As for the size, analysis of variance showed that the model was significant (F-value = 9.54) with a lack of fit not significant relative to the pure error. Among the evaluated independent variables, the influence of individually evaluated variables on PDI have not statistical significance, whereas the interactions of variables AB and AD had big impact on PDI, with *p* values much lower than 0.05 (AB *p* value=.008; AD *p* value<.0001). The use of large amounts of oil has a negative impact on population homogeneity with higher PDI values, which can be ameliorated increasing the surfactant or co-surfactant content in the formulation, as showed in the diagram for these interactions displayed in Figure 2 (I and II, red lines).

#### 4.2.3. Effect of independent variables on zeta potential

**Figure 3.**
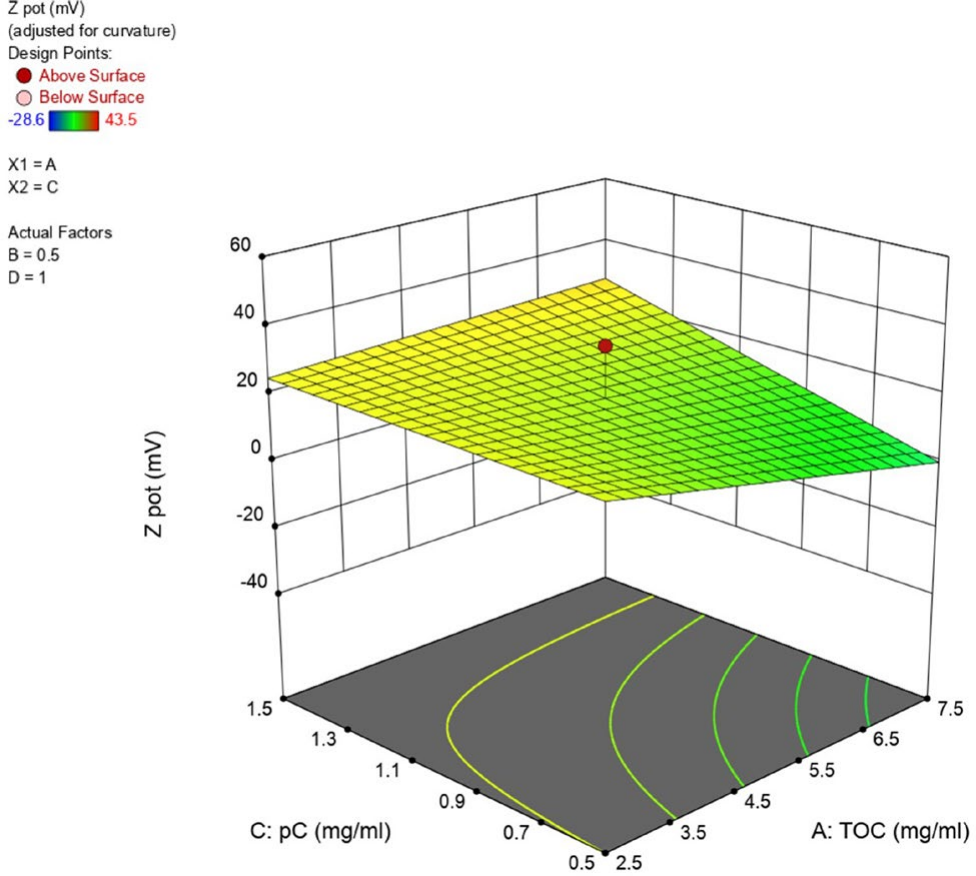
Zeta potential 3D surface diagram for variables A and C interaction.

For zeta potential evaluation, the model was also significant according to the analysis of variance (F-value = 3.43), with a lack of fit not significant relative to the pure error. Among the evaluated independent variables, pC content (variable C) is the only one with statistically significant influence on the zeta potential (*p* value = 0.044) (Figure S2, Supplementary Material), which is slightly influenced by the interactions involving TOC as displayed in the 3D surface diagram in Figure 3. Content in ODA has an impact on zeta potential due to its cationic nature, but it is not significant in the model (*p* value=.18).

#### 4.2.4. Formulations optimization and prediction

After the analysis of data collected from the screening, two optimization runs were carried out introducing in the software several criteria concerning desired ranges for the different parameters. The first optimization criteria included: size in range between 150 and 200 nm, PDI in range between 0.09 and 0.2, and zeta potential in range between +30 and +45 mV. In a second optimization run we maintained these criteria but included a further constrain to maximized TOC content. In the first optimization run we obtained 84 solutions, while we obtained 74 solutions in the second. Confirmation tests were performed by the synthesis of 2 of the predicted compositions in triplicate, encoded as formulation pC-NE_T_ (optimization run 1) and formulation pC-NE_U_ (optimization run 2) respectively. As shown in Table 3, observed responses for both pC-NE_T_ and pC-NE_U_ formulations agreed with predicted values and were within the confidence intervals bounds, showing the suitability of selected DoE model. Formulations with these compositions were used for further characterization.

**Table 3.** Composition of optimized formulations pC-NE_T_ and pC-NE_U_: predicted and experimental values (observed mean) for hydrodynamic size (MDD), PDI and zeta potential (ZP).

Code	Composition	Amount (mg)	Responses	Predicted mean	95% CI low for mean	95% CI high for mean	Observed mean (mean ± SD, *n* = 3)
pC-NE_T_	TOC	2.5	MDD (nm)	199.0	106.9	291.1	184.6 ± 7.5
	ODA	0.62	PDI	0.168	0.117	0.219	0.095 ± 0.006
	T80	0.25	ZP (mV)	+31.8	+15.9	+47.8	+21.7 ± 2.0
	pC	0.5					
pC-NE_U_	TOC	7.5	MDD (nm)	175.6	65.4	285.9	255.9 ± 7.8
	ODA	0.85	PDI	0.108	0.047	0.170	0.131 ± 0.010
	T80	1.75	ZP (mV)	+29.7	+12.3	+47.1	+37.8 ± 1.2
	pC	1.1					

CI: confidence interval bounds

### 4.3. Physicochemical characterization of pC-NE

Three independent batches of pC-NE_T_ and pC-NE_U_ were synthesized as previously described in the experimental section (see Table 3 for summary of physicochemical characterization). Both compositions (pC-NE_T_ and pC-NE_U_) led to NE smaller than 300 nm with very low PDI <0.15. The zeta potential was around +22 mV for formulation pC-NE_T_ and higher in the case of formulation pC-NE_U_, close to +40 mV.

**Figure 4.**
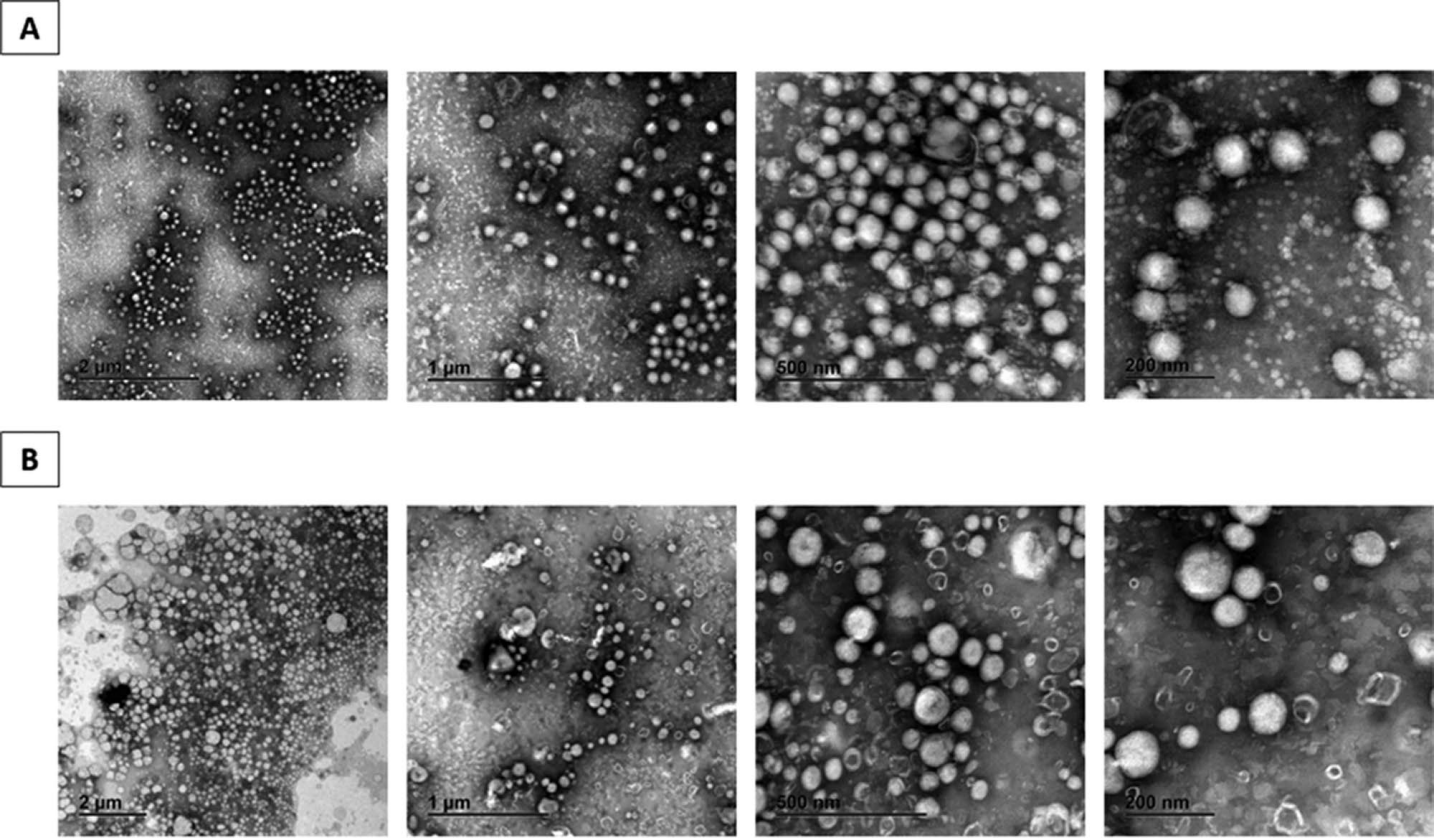
TEM images of pC-NE_T_ (A) and pC-NE_U_ (B) formulations.

The morphological analysis of formulations pC-NE_T_ and pC-NE_U_ was performed by TEM. Both NE showed a spherical shape with a uniform size distribution (Figure 4), without the presence of large aggregates.

Due to its stability behavior (see 4.4), we only determined the effective chemical composition of formulation pC-NE_U,_ through determination of synthesis process yield, EE and loading capacity (Table 4).

**Table 4.** Chemical characterization of blank and pC-loaded NE_U_ formulations (mean ± SD, *n* = 3).

NE identification	Process yield (%)	Entrapment efficiency (%)	Loading capacity (%)
Blank-NE_U_	83.3 ± 4.9	–	–
pC-NE_U_	81.0 ± 5.0	89.7 ± 6.3	14.0 ± 0.4

### 4.4. Stability studies

**Figure 5.**
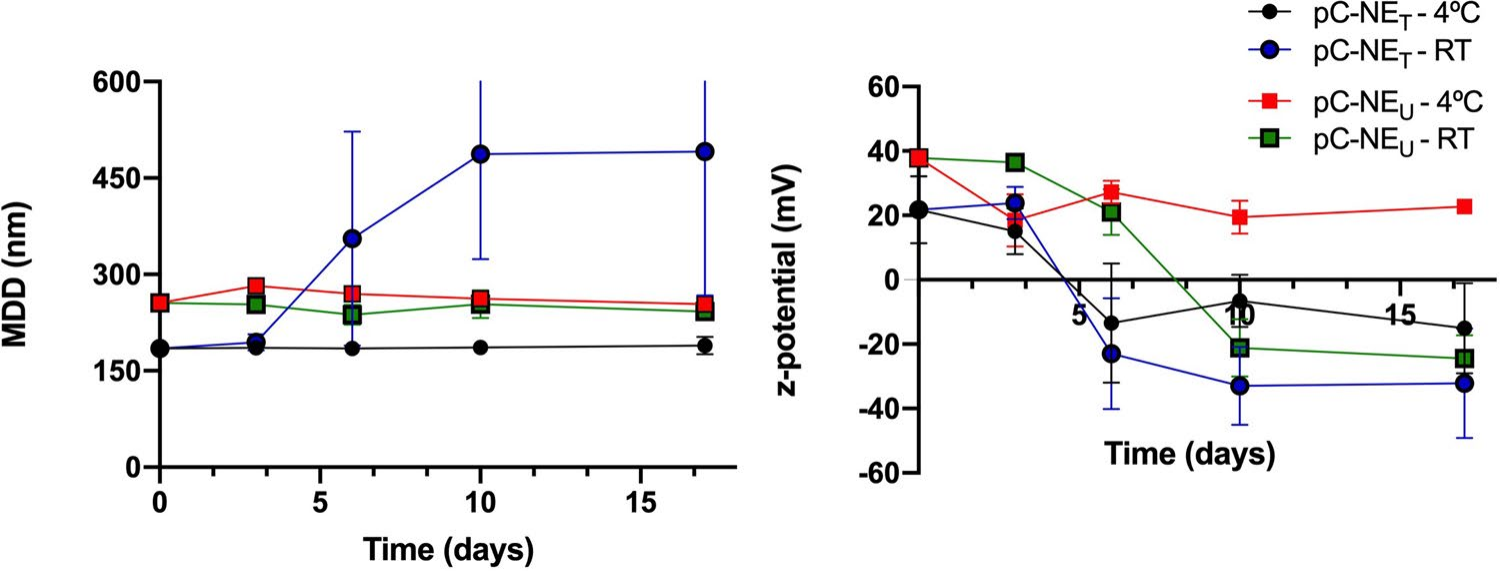
Effect of storage conditions on particle size and zeta potential on pC-NE_T_ and pC-NE_U_ formulations in PBS 1x (mean ± SD, *n* = 3).

The physical stability of both pC-NE_T_ and pC-NE_U_ formulations was evaluated in PBS following storage at 4 °C and RT for 2 weeks. PBS was selected as a relevant buffer for future biological studies. No significant changes in the particle size and PDI of NE were observed except for formulation pC-NE_T_ stored at RT, which increased both parameters over time, with a size close to 500 nm after two weeks. All the NE, except for pC-NE_U_ stored at 4 °C, showed a decrease in zeta potential, reaching negative values after five days (Figure 5). According to the stability results obtained for all the batches at the assessed conditions, pC-NE_U_ formulation was selected as the best pC-NE for the following experiments.

**Figure 6.**
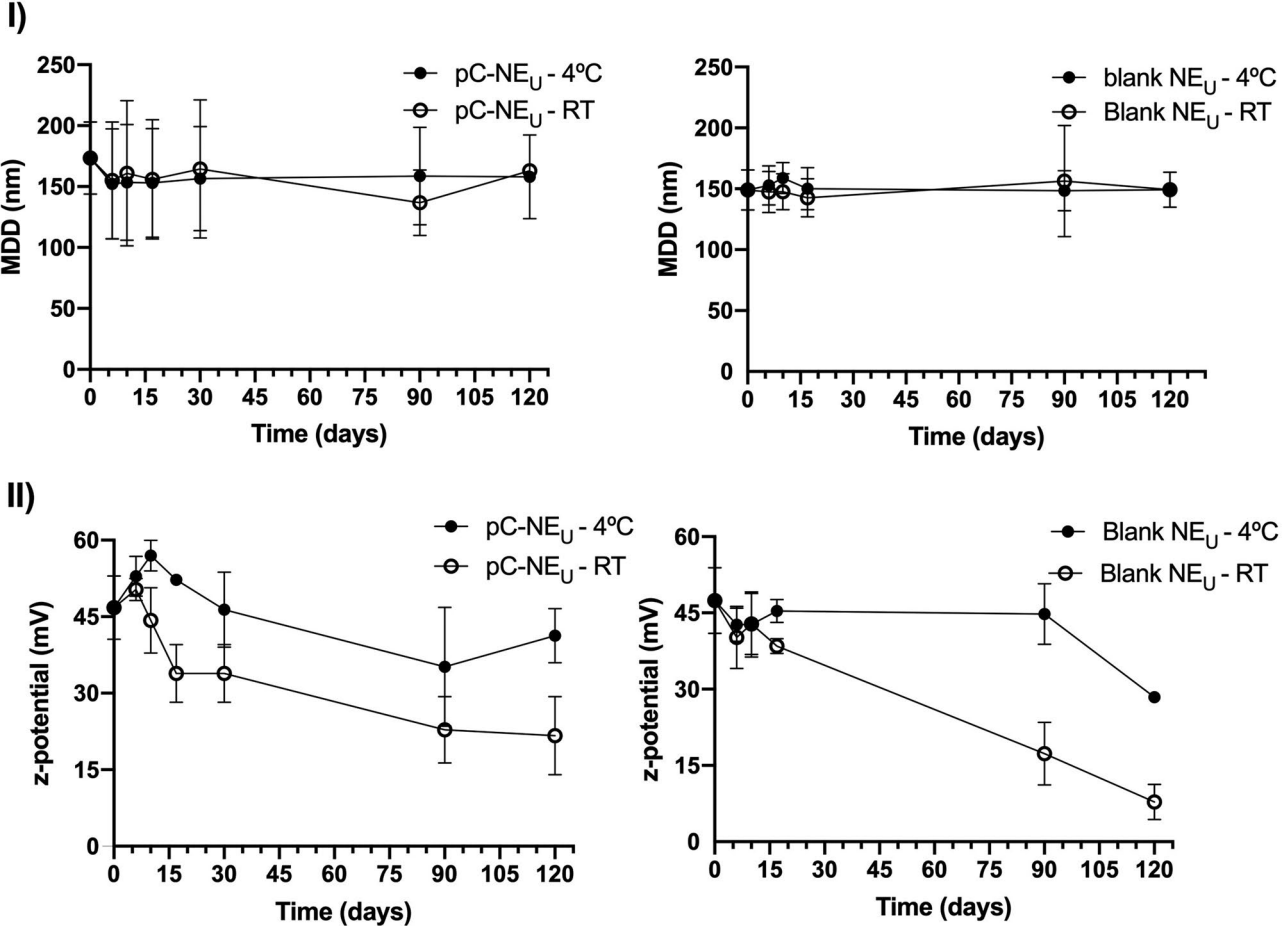
Effect of storage conditions on particle size (I) and zeta potential (II) on pC-NE_U_ and blank NE_U_ formulations in ultrapure water (mean ± SD, *n* = 3).

**Figure 7.**
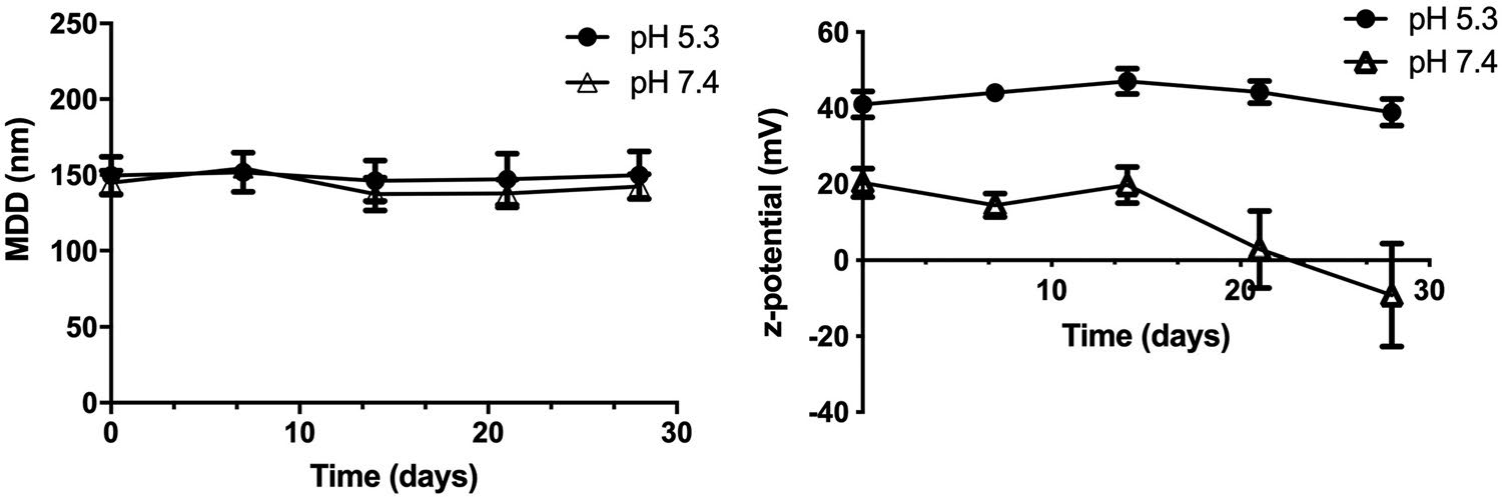
Effect of pH on particle size and zeta potential on pC-NE_U_ formulation (mean ± SD, *n* = 3).

To study the long-term stability of this composition, NE_U_ formulations (both blank and pC-loaded) were synthesized and stored in ultrapure water at 4 °C and RT for 4 months, being evaluated at pre-established time points (Figure 6). Particle size was significantly smaller (159 ± 8 nm) when NE was prepared in ultrapure water instead of PBS. pC-NE_U_ formulation stability was also evaluated under storage in low ionic strength buffers at pH 7.4 and pH 5.3. Figure 7 shows the evolution of hydrodynamic diameter and PDI over four months at those pH values. NE stored at pH 7.4 presented a decrease in zeta potential over time, reaching negative values after 3 weeks. However, no significant changes were observed in particle size and zeta potential at pH 5.3, where NE presented also low PDI over time.

### 4.5. Scalability of the process

The reaction leading to the formation of Blank NE_U_ was subjected to a scale up process, increasing the compounds amount used in the small-scale synthesis up to 10 times. The resulting NE was purified by flow tangential filtration using Vivaflow Cassettes connected to a peristaltic pump (Watson-Marlow 323). We did not observe major changes in initial physicochemical properties (see Supplementary Material, Table S2). The stability behavior of scaled blank NE_U_ stored at both 4 °C and RT during 4 months was comparable to the non-scaled up batches in terms of size, whereas the reduction of zeta potential in the formulations stored at RT was larger in the scaled-up samples (Figure S3, Supplementary Material).

### 4.6. Pc-NE_U_ formulation biodistribution

A fluorescently labeled pC-NE_U_ was developed by adding DID’ oil dye in the organic phase for studying its biodistribution in mice. The physicochemical properties of the fluorescent pC-NE_U_ formulation (DID-pC-NE_U_) were not significantly different to those of non-fluorescent pC-NE_U_ formulation (see Supplementary Material, Table S3). The EE of the dye was very high, close to 100%, confirming the affinity of this kind of dyes for the lipid core of the NE. The stability of fluorescent labeling was studied up to 24 h by incubation of DID-pC-NE_U_ in PBS at 37 °C. No release of fluorescent probe was detected (Figure S4, Supplementary Material).

**Figure 8.**
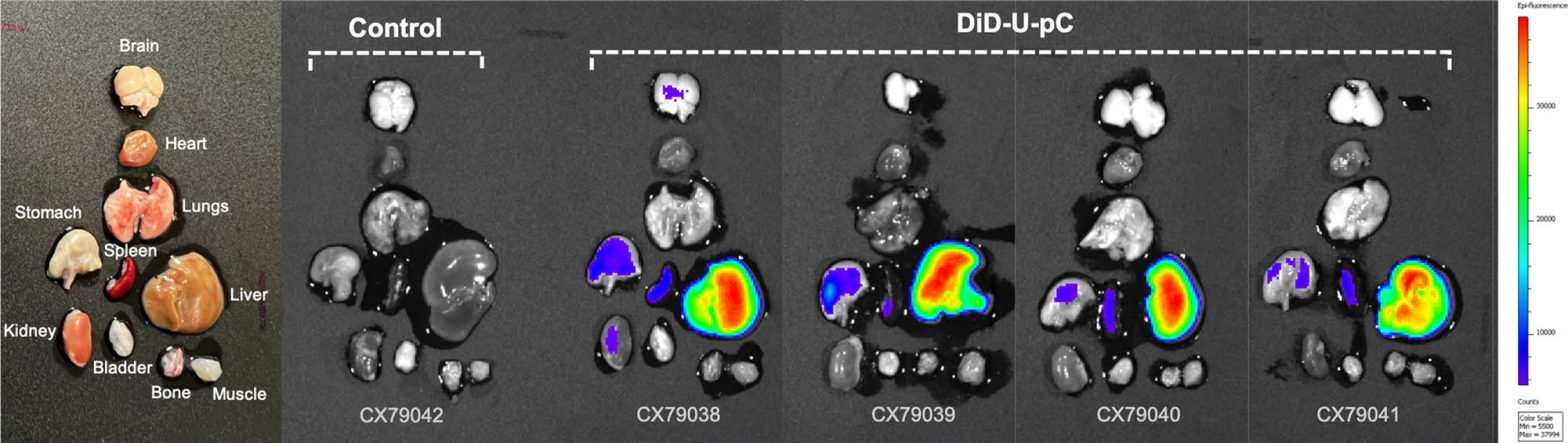
Ex vivo fluorescence imaging of dissected tissues from C57BL/6 mice non-injected (control) and four hours post injection with DID-pC-NE_U_. Fluorescence was adjusted using the non-injected mice as control in order to eliminate the natural autofluorescence given by all tissues.

Four C57BL/6 mice were IV injected with DID-pC-NE_U_; four hours post-injection, mice were sacrificed and after perfusion with 4% formaldehyde the fluorescence from dissected tissues was qualitatively measured (Figure 8). The dissected organs from a non-injected C57BL/6 mouse were used as control to eliminate the autofluorescence given by all tissues, thus leaving only the fluorescence produced by the injected NE. Live images of whole animals were not taken as they suffer from low light penetration into biological tissues. We found that DID-pC-NE_U_ formulation was predominantly accumulated in the liver, with minimal accumulation in spleen, stomach, and kidneys.

## 5. Discussion

As previously described, the rational selection of lipids and surfactants/co-surfactants plays a critical role in the formation of small and stable NE (Gupta et al., 2016). We selected TOC as liquid lipid for NE due to its excellent solubilizing capacity for many poorly water-soluble drug substances (Nielsen et al., 2001). Additionally, ODA was selected as co-surfactant because of its cationic nature. It has been described that cationic compounds provide several advantages for drug delivery applications, such as high efficiency in delivery of nanomaterials in cells, wide availability, cost effectiveness, chemical and physical stability, and biocompatibility, among others (Young et al., 2020; Daull et al., 2014; Khachane et al., 2015). We also studied the effect of using two surfactants, T20 and T80, on NE development. The surfactant plays an important role in the final selection of NE formulations, since the emulsifier decreases the interfacial tension between the two phases, aiding in the dispersion process required to form the product (Handa et al., 2021). As shown in Table 2, the smallest particle size (240 nm) and PDI value (0.2) were obtained using T80 as surfactant. In drug delivery applications using lipid-based carriers, PDI value lower than 0.3 indicates homogeneous system (Danaei et al., 2018). Accordingly, T80 provided more homogeneous NE, thus more stable NE. The different behavior observed for both surfactants may be explained by their chemical structure. Both polysorbates, with a common backbone, differ in the fatty acid sidechains, oleic acid in the case of T80, lauric acid for T20. The fatty acid chain length and degree of unsaturation in the surfactant influence particle size, as shorter chain length confers rigidity to the fatty acid, resulting in larger particles with lower curvature. Moreover, the higher the number of unsaturation in the hydrocarbon chain, the more flexible is the chain and smaller the particles obtained (Eh Suk et al., 2020). Smaller particle size was obtained for NE containing T80, probably due to the bend and kink effect at the double bond of monooleate, which increased the curvature of NE droplets (Saberi et al., 2013). It is also worth noting that not only the type, but also the surfactant concentration influenced the final size of NE. Increasing T20 concentration from 0.1 to 10 mg/mL caused a drastic particle size decrease, probably due to the lack of stabilizing surfactant that triggered particles aggregation at the lowest concentrations. However, increase in T80 concentration resulted in slightly larger particles and higher polydispersity. This behavior may be explained by a reduced diffusion rate of the molecules in the sample caused by an increased viscosity of the continuous phase. Accordingly, based on the size and PDI obtained for all the synthesized batches, T80 was selected as the best surfactant for stabilizing the NE.

DoE is a popular and widely used research approach for determining the critical factors affecting processes, such as nanoparticles production. After the first screening for the selection of T80 as the most adequate surfactant, a FFD was used for evaluation of NE composition’s effect on main responses, like particle size, PDI, and zeta potential, which have been described to be mainly influenced by lipids and surfactants composition, as well by conditions for synthesis (Tavares Luiz et al., 2021). As previously described, the selection of FFD was based on the possibility it offers to explore a maximum number of variables without a lack of main effects data but with less experimental effort compared to full factorial designs (Kuncahyo et al., 2019). Synthesizing 19 prototypes with different composition during the screening phase (Table S1, Supplementary Material) we obtained the needed information to identify the critical factors influencing NE physicochemical properties as drug carrier for pC, used here as a model hydrophobic drug. Regarding NE size, the only variable with statistical significance influence was TOC content. The influence of lipid concentration on particle size has been previously described for other lipid nanoparticles (Schubert & Müller-Goymann, 2003; Sarheed et al., 2020). On the other hand, PDI was influenced by the interactions of two variables, TOC-T80 (interaction AB) and TOC-ODA (interaction AD) (Figure 2). The effect of these interactions could be explained because the type and concentration of surfactant and co-surfactant, like ODA, is important in stabilizing emulsions and preventing aggregation of the droplets, thus maintaining a low PDI (Sharma et al., 2016). Finally, zeta potential is significantly influenced by pC content (variable C), probably due to the localization of this cationic moiety at the interface, as previously observed for other cationic compounds (Chinigò et al., 2022). This is particularly interesting as positive surface charge may be desirable to increase the interaction of the nanocarrier with cellular membranes (Mamusa et al., 2017; Nazarenus et al., 2014; Åberg et al., 2021).

The use of DoE allowed us to make a prediction on the composition that will show optimized physicochemical properties, having the possibility of somehow modulating their biological behavior (Danaei et al., 2018). Both optimized pC-NE_T_ and pC-NE_U_ formulations showed particle size smaller than 300 nm with very low PDI < 0.15, which indicates the homogeneity of size distribution. The different behavior in zeta potential observed for both formulations may be explained by their composition, as formulation pC-NE_U_ contains higher concentration of cationic compounds (ODA and pC), thus showing a higher zeta potential (Wang & Keller, 2009).

Based on TEM images (Figure 4), both NE showed a spherical shape with a uniform size distribution, which is consistent with the size determined by PCS. Furthermore, no large aggregates were observed, indicating homogeneous particle populations, corresponding with low PDI obtained by PCS. However, stability studies at 4 °C and RT revealed all the NE, except for refrigerated pC-NE_U_, reached negative values after 5 days, which indicates that storage at 4 °C is more appropriate to keep the initial properties for longer time. Differences found in stability behavior between both compositions may be related to the different zeta potential showed by formulation pC-NE_T_ and pC-NE_U_. The surface charge of particles plays an important role in their physical stability as it may influence the rate of aggregation and fusion among particles. It has been suggested that full electrostatic stabilization requires a zeta potential higher than 30 mV in absolute values, being particles with potentials within that range more unstable and with higher tendency to flocculate. Although it is not the unique parameter influencing colloidal stability of nanosuspensions (Wang & Keller, 2009), in the case of pC-NE_T_ (+22 mV) seems to have big impact in the showed short-term stability in PBS. Additionally, it is worth noting particle size of pC-NE_U_ was significantly smaller when NE was prepared in ultrapure water (159 nm) instead of PBS (256 nm). Differences found in physicochemical properties when pC-NE_U_ was synthesized in different aqueous phases could be explained by the nature of intermolecular interaction established. According to DLVO theory, in ultrapure water the electrostatic repulsion of NE is higher than van der Waals forces, thus they can remain dispersed during long time. However, due to the high ionic strength of PBS, the electrical double layer is compressed and the electrostatic repulsion decreases inducing the size increase of the colloidal system (Moore et al., 2015). No significant changes in particle size were observed for both NE when stored in ultrapure water at 4 °C and RT, which is not surprisingly due to the low ionic strength of water compared to PBS. Additionally, the high EE and loading capacity of pC in pC-NE_U_ (Table 4) confirmed the ability of the designed NE to incorporate the hydrophobic model compound in an efficient manner. The yield of the synthesis reaction for pC-NE_U_ was high as well, demonstrating the suitability of the solvent injection technique as synthesis method for the development of these NE.

Furthermore, pH stability is an important parameter for determining the susceptibility of the drug formulation to hydrolysis when in solution or suspension (Blessy et al., 2014). pC-NE_U_ stored at pH 7.4 presented a decrease in zeta potential over time, reaching negative values after 3 weeks, which indicates that modifications in surface charge depend on pH of surrounding environment. However, no significant changes were observed in particle size and zeta potential at pH 5.3, where pC-NE_U_ presented also low PDI over time, indicating pC-NE_U_ formulation is quite stable at acidic pH. Accordingly, pC-NE_U_ may be potential candidate to be used as drug delivery system for targeting disease environments in which acidic pH can be found, such tumors or infections sites (Percival et al., 2014; Boedtkjer & Pedersen, 2020).

Finally, scale-up studies showed no major change in initial physicochemical properties of blank NE_U_. One of the keys to effective clinical use of nanoparticles is scaling up the nanoformulation process to manufacture quality-controlled large-batch nanoparticles (Paliwal et al., 2014). Here blank NE_U_ was successfully scaled up, indicating the suitability of both raw materials and scale-up synthesis protocol for the reproducible synthesis of large-batch NE. Additionally, physicochemical properties of blank NE_U_ stored at both 4 °C and RT were comparable to the non-scaled up batches, confirming a preserved functionality of blank NE_U_.

The biodistribution studies were based on DID-pC-NE_U_ analysis in mice. The EE of the dye was very high, close to 100%, confirming the affinity of this kind of dyes for the lipid core of the NE. Particle uptake by cells of the reticuloendothelial system (RES) could explain the high fluorescence recorded from the liver (Kumar et al., 2010), which also play an important role in the elimination and detoxification of different metabolites from the bloodstream, as well as lipid processing (Mannucci et al., 2020). Similar biodistribution pattern has been described for NE ranging from 70 to 200 nm, as size has a crucial role in the *in vivo* fate of NE (Fan et al., 2020). Moreover, Busman et al. have described that particle size influences accumulation in stomach after iv administration, finding the maximum accumulation in this organ for 150 nm NE compared to smaller NE (Busmann & Lucas, 2022), which agrees with our findings. Nevertheless, this biodistribution profile could be modulated changing physicochemical properties of the nanocarriers, such as size and surface composition (Hirsjärvi et al., 2013).

## 6. Conclusions

Cationic NE were successfully developed for the encapsulation of the hydrophobic model drug pC. The use of DoE approach appears as a useful tool for obtaining NE with desired physicochemical properties with minimized experimental effort. One of the optimized compositions, pC-NE_U_, led to a NE with small hydrodynamic size (<200 nm), narrow size distribution (PDI < 0.150) and high positive zeta potential (+40 mV) which showed good stability profiles at different conditions of pH and temperature, and also after scaling up the synthesis process, which is highly desirable from a pharmaceutical industry point of view, ensuring an easier translation process. Moreover, pC-NE_U_ showed high entrapment capacity of pC, corroborating our initial hypothesis of the potential of NE, if properly designed, as a drug delivery system for hydrophobic active compounds, and the biodistribution profile obtained is in line with those found by other authors for NE with similar size.

In conclusion, the use of DoE allows for a rational design of drug delivery platforms. The simple composition of pC-NE_U_, its easily prepared and the properties here showed make it a promising template for delivery of hydrophobic compounds, which may require an easy reformulation process depending on the drug.

## Supplementary Material

Supplemental Material
