# Supplementary material for: A quality by design approach for the synthesis of palmitoyl-L-carnitine-loaded nanoemulsions as drug delivery systems
Source: Drug Deliv. 2023 Feb 19;30(1):2179128. doi: 10.1080/10717544.2023.2179128 (PMC10184586; doi:10.1080/10717544.2023.2179128)
Supplement: Supplemental Material [file IDRD_A_2179128_SM0308.docx]

**Supplementary material**

**A quality by design approach for the synthesis of palmitoyl-L-carnitine loaded nanoemulsions as drug delivery systems.**

Eva Arroyo-Urea^a^, María Muñoz-Hernando^a^, Marta Leo-Barriga^a^, Fernando Herranz^a,b,c^*, Ana González-Paredes^a,c^*

^a^Nanomedicine and Molecular Imaging group, Instituto de Química Médica-CSIC, Juan de la Cierva, 3- 28006 Madrid (Spain)

^b^Centro de Investigación Biomédica en Red de Enfermedades Respiratorias (CIBERES)

^c^Conexión Nanomedicina-CSIC

**Corresponding authors:* Ana González-Paredes & Fernando Herranz

E-mail adresses: [ana.gonzalez@iqm.csic.es](mailto:ana.gonzalez@iqm.csic.es), [fherranz@iqm.csic.es](mailto:fherranz@iqm.csic.es)

**1. Supplementary methods**

## 1.1. Sample preparation for determination of entrapment efficiency of pC-NE

The concentration of pC in pC-NE_U_ formulation was determined before and after purification process after synthesis through HPLC-RID analysis, as described in the main text body. A calibration curve was obtained by dissolving pC in methanol: chloroform mixture (3:2 v/v). This solvent mixture was also used for disrupting pC-NE for HPLC samples preparation by diluting pC-NE 1:5 (v/v) in methanol: chloroform (3:2 v/v). Finally, the volume was completed with eluent in order to have a final dilution of the sample 1:50 (v/v). The diluted samples were then centrifuged for 10 minutes 10000 rpm to ensure NE breaking and filtered (0.2 µm RC) before analysis.

**2. Supplementary results**

2.1. Fractional factorial design

The fractional factorial design that was selected as model required the synthesis and characterization of nineteen formulations of pC-NE, encoded A-S. The composition of these 19 formulations is displayed in Table S1, whereas the histograms with the corresponding physico-chemical properties are displayed in Figure S1. Most of the samples showed small hydrodynamic sizes (180-300 nm), narrow size distribution (PDI<0.3) and positive zeta potential.

**Table S1.** Composition of pC-NE formulations synthesized during screening phase

| Formulation code | **Composition (mg/mL)** | | | |
| --- | --- | --- | --- | --- |
|  | **TOC** | **ODA** | **pC** | **T80** |
| pC-NE_A_ | 5 | 0.5 | 1 | 1 |
| pC-NE_B_ | 2.5 | 0.85 | 1.5 | 0.25 |
| pC-NE_C_ | 7.5 | 0.15 | 0.5 | 1.75 |
| pC-NE_D_ | 7.5 | 0.15 | 1.5 | 0.25 |
| pC-NE_E_ | 2.5 | 0.15 | 1.5 | 1.75 |
| pC-NE_F_ | 7.5 | 0.85 | 0.5 | 0.25 |
| pC-NE_G_ | 5 | 0.5 | 1 | 1 |
| pC-NE_H_ | 7.5 | 0.15 | 0.5 | 1.75 |
| pC-NE_I_ | 5 | 0.5 | 1 | 1 |
| pC-NE_J_ | 7.5 | 0.85 | 0.5 | 0.25 |
| pC-NE_K_ | 7.5 | 0.85 | 1.5 | 1.75 |
| pC-NE_L_ | 2.5 | 0.15 | 1.5 | 1.75 |
| pC-NE_M_ | 2.5 | 0.85 | 0.5 | 1.75 |
| pC-NE_N_ | 7.5 | 0.15 | 1.5 | 0.25 |
| pC-NE_O_ | 2.5 | 0.15 | 0.5 | 0.25 |
| pC-NE_P_ | 2.5 | 0.15 | 0.5 | 0.25 |
| pC-NE_Q_ | 2.5 | 0.85 | 1.5 | 0.25 |
| pC-NE_R_ | 7.5 | 0.85 | 1.5 | 1.75 |
| pC-NE_S_ | 2.5 | 0.85 | 0.5 | 1.75 |

**
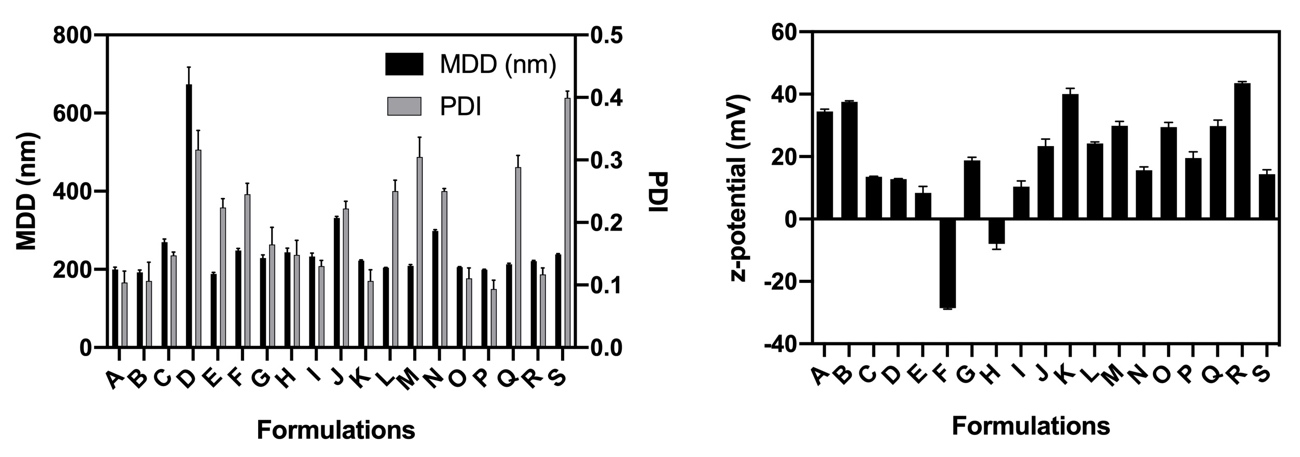
Figure S1**. Particle size, polydispersity index and zeta potential of pC-NE formulations. MDD: Mean droplet diameter; PDI: polydispersity index, z-potential: Zeta potential

2.2. Analysis of critical variables by DoE: effect of independent variables on zeta potential

Among the evaluated independent variables influencing zeta potential, pC content (variable C) is the only one with statistically significant influence in the zeta potential (p-value=0.044). Figure S2 displays the effect of the 4 individual variables on zeta potential.


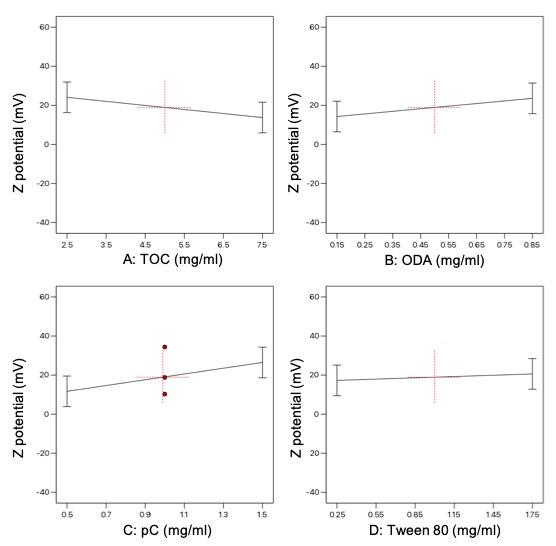


**Figure S2**. Main effect plot showing the effect of individual variables on zeta potential.

2.3. Characterization of scaled up blank-NE_U_ formulation

Table S2 summarizes the physicochemical properties of Blank NE_U_ formulation at two production scales: lab batch and scaled up production, whereas Figure S3 represents it stability behavior during storage at 4ºC and RT in water during 120 days.

**Table S2**. Physico-chemical characterization of Blank NE_U_ formulations synthesized at two different scales (lab batch and scaled up batch): hydrodynamic size (MDD), PDI and zeta potential (ZP) (mean ±SD, n=3)

| Code | MDD (nm) | PDI | ZP (mV) |
| --- | --- | --- | --- |
| Blank-NE_U_ (lab batch) | 149.1 ± 16.6 | 0.124 ± 0.015 | + 47.4 ± 6.5 |
| Blank-NE_U_ (scaled up) | 140.0 ± 12.0 | 0.175 ± 0.050 | + 46.8 ± 1.0 |

**
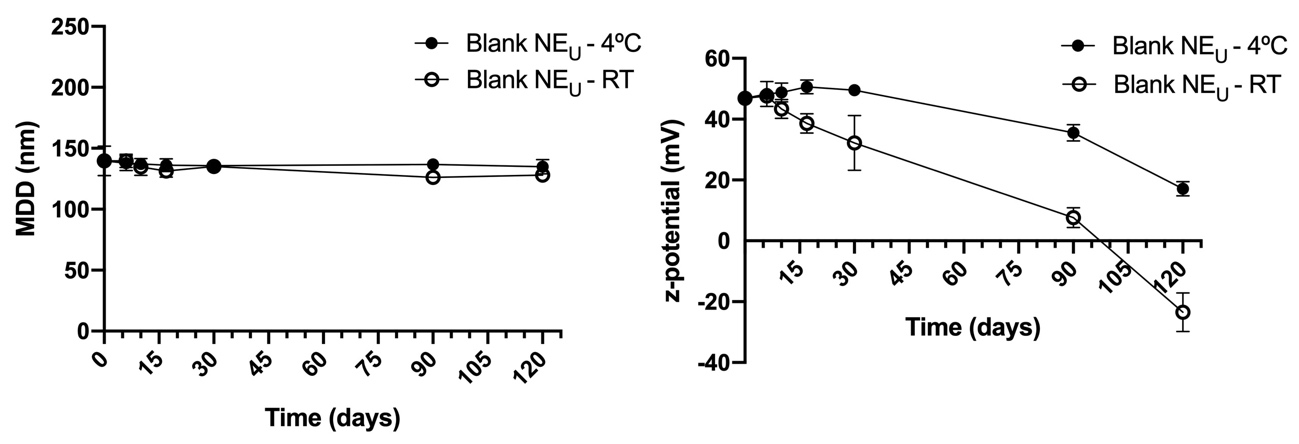
**

**Figure S3**. Effect of storage conditions on particle size and zeta potential on Blank NE_U_ formulations scaled up and stored in ultrapure water (mean±SD, n=3).

2.4. Characterization of DID-pC-NE_U_ formulation

Table S3 summarizes the physicochemical properties of pC- NE_U_, both non-fluorescent and DID loaded (DID-pC-NE_U_) whereas figure S4 displays release profile of the fluorescent dye after DID-pC-NE_U_ incubation in PBS 1X at 37ºC during 24h.

**Table S3**. Physico-chemical characterization of non-fluorescent pC- NE_U_ and DID-pC-NE_U_: hydrodynamic size (MDD), PDI and zeta potential (ZP) (mean ±SD, n=3)

| Code | MDD (nm) | PDI | ZP (mV) |
| --- | --- | --- | --- |
| pC-NE_U_ | 158.7 ± 8.0 | 0.149 ± 0.024 | +45.9 ± 6.2 |
| DID-pC-NE_U_ | 100.1 ± 6.0 | 0.159 ± 0.037 | +31.4 ± 6.1 |


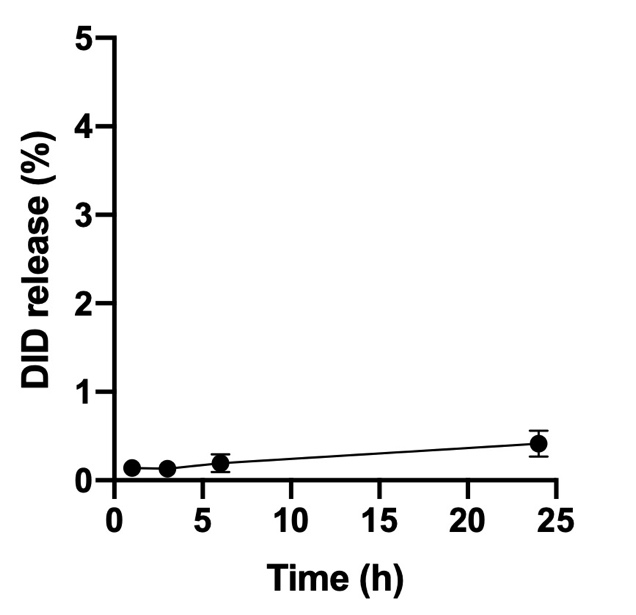


**Figure S4.** Release profile of DID dye from DID-pC-NE_U_ upon incubation in PBS 1X at 37ºC during 24h (mean ±SD, n=6)
